# Bilateral Adrenal Myelolipomas in a Female Patient With Undiagnosed Non-classic Congenital Adrenal Hyperplasia

**DOI:** 10.7759/cureus.35017

**Published:** 2023-02-15

**Authors:** Bhavana R Vemula, Omolola B Olajide, Yewande Adepoju

**Affiliations:** 1 Endocrinology, Diabetes, and Metabolism, Marshall University Joan C. Edwards School of Medicine, Huntington, USA; 2 Endocrinology, Diabetes, and Metabolism, Oroville Hospital, Oroville, USA

**Keywords:** female with non-classic cah, bilateral adrenal myelolipomas, myelolipomas, congenital adrenal hyperplasia, non-classic cah

## Abstract

Non-classic congenital adrenal hyperplasia (CAH) usually presents later in life with signs of androgen excess but may also be diagnosed after the detection of an incidental adrenal myelolipoma. This is a patient with previously undiagnosed CAH who presented to the emergency department with chest discomfort and palpitations. A computed tomography (CT) scan of the chest done to rule out pulmonary embolism showed bilateral large adrenal myelolipomas. She also had evidence of marked hirsutism on examination, which prompted further workup, and her laboratory data was in keeping with CAH. Further management was unable to be pursued due to the patient’s poor compliance, and she was subsequently lost to follow-up. Chronic exposure of the adrenal glands to high adrenocorticotropic hormone (ACTH) levels increases the risk of developing myelolipomas. CAH needs to be considered as a diagnosis in the evaluation of incidental adrenal myelolipomas.

## Introduction

Congenital adrenal hyperplasia (CAH) is an autosomal recessive disorder involving enzymatic deficiencies leading to decreased cortisol production and subsequent overproduction of adrenocorticotropic hormone (ACTH). This results in chronic overstimulation of the adrenal cortex and subsequent androgen excess in some cases [1]. Depending on the severity of the enzyme deficiency, patients can present as early as the newborn stage to as late as the sixth or seventh decade of life. Female patients are usually diagnosed earlier when compared to male patients due to the symptoms of virilization. Adrenal myelolipomas are benign adrenal tumors that are usually incidental findings on imaging [2]. They could occasionally be the initial presentation of CAH [3]. This is a case of a female patient with undiagnosed CAH who presented with bilateral adrenal myelolipomas in her sixth decade of life.

## Case presentation

A 68-year-old female patient presented to the emergency department with symptoms of chest discomfort, palpitations, and poor living conditions. She has a known history of untreated schizophrenia and anxiety. A computed tomography (CT) of the chest was done to rule out pulmonary embolism and showed bilateral adrenal masses and enlargement of the right thyroid. Endocrinology was consulted for the adrenal nodules and hirsutism.

The patient was a poor historian, and limited history was available. She had been living alone for many years as most of her close family members were deceased. According to the nursing staff and report from adult services, the patient has a brother who reported that she was labelled “hermaphrodite” and her mother shielded her away from the community. The patient stated that she had periods between the ages of 14 and 16 years and none since then. She denied any changes in her hair growth, skin, weight, or voice. However, she had a long beard shaved by the nursing staff on presentation. The patient was not sexually active.

She denied having any surgeries, smoking, and alcohol or drug use. Her family history is not known. On admission, her temperature was 98.6°F, pulse was 105 beats per minute, blood pressure was 145/111 mmHg, respiratory rate was 18 breaths per minute, and oxygen saturation was 96% on room air. On examination, she was awake, alert, and oriented to self and had a masculine appearance with scanty hair on the scalp, bald spots anteriorly, shaved facial hair, excessive hair on the extremities and on the back, poor dentition, and well-developed breasts. On genitourinary examination, she had pubic hair extending to the umbilicus, excess labial folds, no clitoromegaly, small vaginal opening, and no palpable testicular tissue.

**Figure 1:**
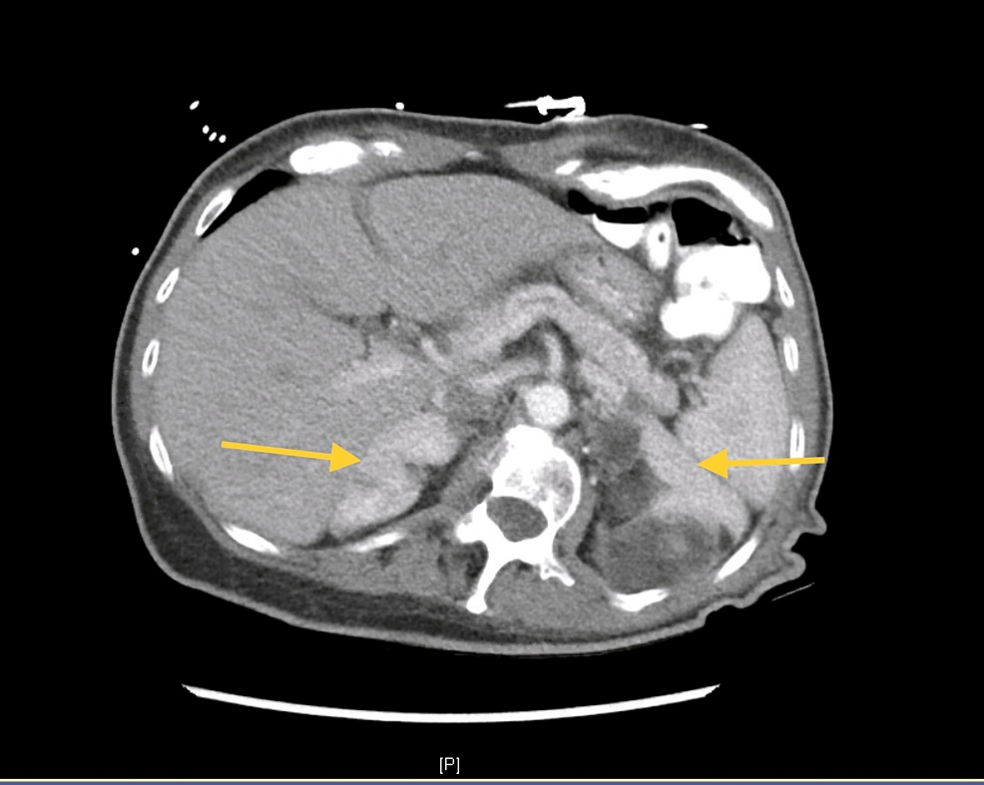
CT of the abdomen with contrast showing bilateral adrenal myelolipomas (axial view) CT: computed tomography

**Figure 2:**
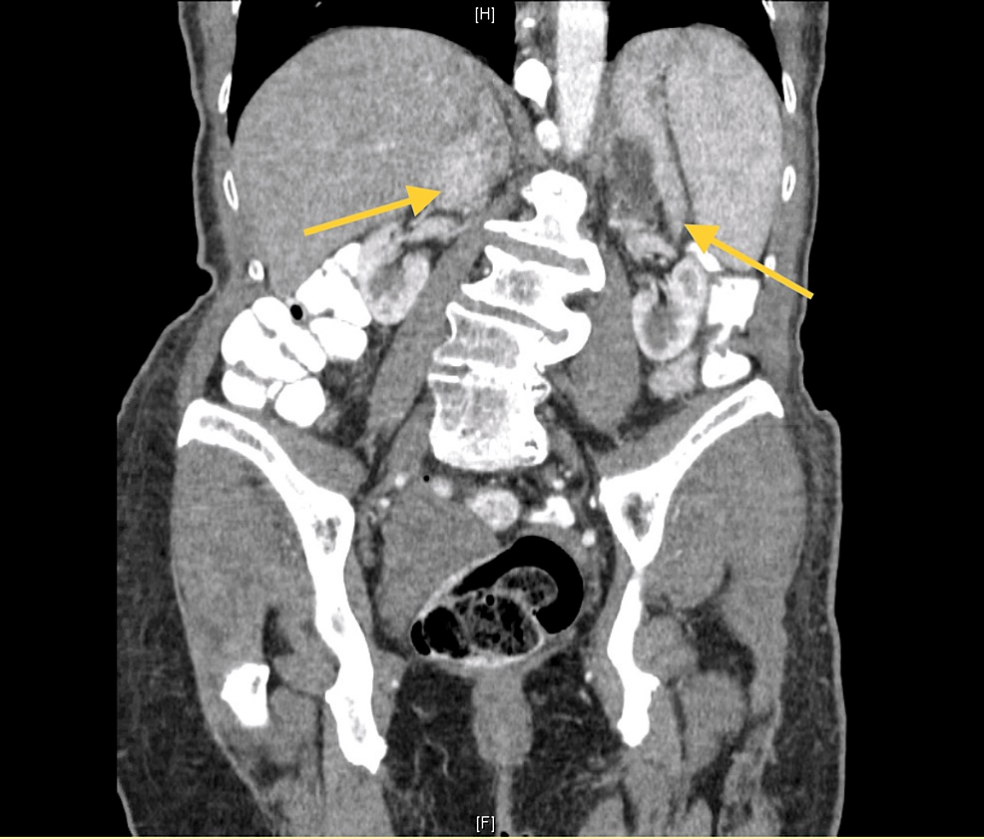
CT of the abdomen with contrast showing bilateral adrenal myelolipomas (coronal view) CT: computed tomography

Laboratory results are shown in Table 1. CT of the abdomen with contrast (Figures 1-2) showed bilateral adrenal nodules. The left nodule was 6.6×9.7×10.5 cm, and the right nodule was 3×7.6×6.8 cm with variable attenuation, 45 Hounsfield units (HU) in the areas of fat. Heterogenous attenuation noted in the nodules is suggestive of myelolipoma. Pelvic ultrasound showed the uterus with a small simple cystic area. There was no evidence of adnexal or pelvic mass. A diagnosis of CAH was made based on the clinical picture and her laboratory data. The patient was discharged to a nursing facility. She came for a follow-up visit in the endocrinology clinic and had a dual-energy X-ray absorptiometry (DXA) scan, which showed osteoporosis with lowest bone mineral density of 0.685 g/cm^2^ at the right femoral neck with T score of -2.5 and Z score of -0.9. Alendronate therapy was initiated. Steroid therapy was not started as there was a high risk of noncompliance. Adrenalectomy was also not considered for the same reason. She was subsequently lost to follow-up.

**Table 1:** Laboratory results LH, luteinizing hormone; FSH, follicle-stimulating hormone; DST, dexamethasone suppression test; ACTH, adrenocorticotropic hormone; DHEA-S, dehydroepiandrosterone sulfate; SHBG, sex hormone-binding globulin; IGF-1, insulin-like growth factor 1

Laboratory parameters	Patient results	Normal values	Laboratory parameters	Patient results	Normal values
Prolactin	15	1.9-25 ng/ml	Androstenedione	2858	17-99 ng/dL
Testosterone	1195	60-80 ng/ml	17-Alpha hydroxyprogesterone	25018	Follicular phase, 15-70 ng/dL; luteal phase, 25-290 ng/dL
LH	<0.2	Postmenopausal: 7.7-58.5 mIU/ml	Plasma metanephrines	11	0-62 pg/ml
FSH	<0.2	Postmenopausal: 25.8-134.8 mIU/ml	Plasma normetanephrines	324	0-874 pg/ml
Cortisol after 1 mg DST	2.8	<1.8 ug/dL	Plasma epinephrine	<15	0-62 pg/ml
Midnight salivary cortisol	0.093	0-0.94 nmol/L	Plasma dopamine	<30	0-48 pg/ml
ACTH	266.7	7.2-63.3 pg/ml	24-hour urine: epinephrines	<2	0-20 ug/24 hours
DHEA-S	353.2	20.4-186.6 ug/dL	24-hour urine: norepinephrine	36	0-135 ug/24 hours
SHBG	90	17.3-125 nmol/L	24-hour urine: dopamine	183	0-510 ug/24 hours
IGF-1	122	38-163 ng/ml	24-hour urine: metanephrine	31	45-290 U/24 hours
			24-hour urine: normetanephrine	248	82-500 U/24 hours

## Discussion

CAH is an autosomal recessive disorder with an incidence of approximately 1:15000 worldwide [1]. Various enzymes involved in steroidogenesis could be affected, among which 21-hydroxylase enzyme deficiency is the most common type, and depending on the extent of the defect, clinical characteristics vary in severity. There are two subtypes of 21-hydroxylase deficiency: the classic form that involves salt wasting and virilization and the milder non-classic form [2,3].

Almost all cases of the classic form of 21-hydroxylase enzyme deficiency are diagnosed in the neonatal period due to the availability of neonatal screening tests over the last few decades [2]. Due to the deficiency of both aldosterone and cortisol, treatment with steroids is essential for survival [3]. In less severe non-classic forms, there is 20%-70% of enzyme activity not detected by neonatal screening tests, and diagnosis can be delayed as the symptoms are milder [2]. Female patients are usually diagnosed earlier due to virilization. However, male patients could present late with precocious puberty or infertility [3]. Our patient was a female who was diagnosed in the sixth decade of life, which is highly unusual.

Myelolipomas were first described in 1905 and are the second most common benign adrenal tumors after adrenocortical adenoma [4]. Adrenal myelolipomas are benign tumors of the adrenal cortex composed of fat tissue and mixed myeloid and erythroid tissue [1]. Most are diagnosed incidentally with distinct findings on imaging like in our patient. Patients with CAH have a high prevalence of adrenal tumors, particularly myelolipomas. Those with myelolipomas had a high frequency of late-diagnosed or poorly controlled CAH [5]. In a literature review, 10% of 440 patients with adrenal myelolipomas had CAH [6]. The etiology is not well-understood, but it is felt that chronic ACTH stimulation may play a role in myelolipoma development especially with untreated or undiagnosed CAH like our patient [1,6]. Myelolipomas are mostly unilateral and asymptomatic but could also be bilateral in location [1]. Bilateral adenomas usually present with larger size and symptoms of mass effect and are more commonly seen in patients with CAH [7]. In a meta-analysis among the patients with CAH and adrenal myelolipoma, 46% were asymptomatic, and 33% were symptomatic with abdominal or flank pain [6].

Patients could also present with acute rupture, hemorrhage, and necrosis of adrenal myelolipomas [8,9]. Very rarely, these are associated with excess hormone-producing adrenal cortical adenomas [7]. Hormonal evaluation is indicated as part of the workup for incidental adrenal myelolipomas with suspected hormone excess: 1 mg overnight dexamethasone suppression test; aldosterone, renin, and metanephrine levels; and 17-alpha hydroxyprogesterone levels for suspected CAH [2]. Due to the high accuracy of imaging features, which include variable attenuation caused by mixed fat and myeloid tissue, adrenal biopsy is usually not recommended [2]. In patients with CAH, glucocorticoid treatment may lead to a decrease in the size of the adrenal myelolipomas [10]. Generally, these masses are managed conservatively with surgery considered for symptomatic myelolipomas and for asymptomatic ones larger than 7 cm in diameter [11]. Bilateral adrenalectomy is preferred in patients with uncontrolled CAH or increasing size of the masses or complications such as acute hemorrhage or ipsilateral adrenal hormonal excess [2,12]. However, patients will need to be on steroid replacement therapy for life after bilateral adrenalectomy.

## Conclusions

Non-classic CAH is a rare condition that is not detected on neonatal screening tests and thus can present very late with unilateral or bilateral myelolipomas. It is especially important for adult endocrinologists to be aware of this fact and the need to screen patients with adrenal incidental myelolipomas for non-classic CAH. This will help ensure early diagnosis and intervention.
